# Beef Tea

**Published:** 1886-03

**Authors:** 


**Beef Tea.**—The valuation by most persons outside of the medical profession, and by many within it, of beef tea or its analogues, the various solutions most of the extracts and the expressed juice of meat, is a delusion and a snare which has led to the loss of many lives by starvation. The quantity of nutritive material in these preparations is insignificant or *nil*, and it is vastly important that they should be reckoned as of little or no value, except as conducive indirectly to nutrition by acting as stimulants for the secretion of the digestive fluids or as vehicles for the introductions of nutritive substances. Furthermore, it is to be considered that water and pressure not only fail to extract the alimentary principles from meat but the extrementitious principles, or the products of destructive assimilation, are thereby extracted. A few years ago, a German experimenter declared that he produced fatal toxœmia in dogs by feeding them with this popular article of diet."—*Dr. Flint.*

